# The kinematics of upper extremity reaching: a reliability study on people with and without shoulder impingement syndrome

**DOI:** 10.1186/1758-2555-2-8

**Published:** 2010-03-23

**Authors:** Jean-Sébastien Roy, Hélène Moffet, Bradford J McFadyen, Joy C MacDermid

**Affiliations:** 1 School of Rehabilitation Science, McMaster University, Hamilton, Ontario L8S 1C7, Canada; 2 Centre for Interdisciplinary Research in Rehabilitation and Social Integration, Quebec City, Quebec, Canada; 3 Department of Rehabilitation, Faculty of Medicine, Laval University, Quebec City, Quebec, Canada; 4 Hand and Upper Limb Centre, St. Joseph's Health Centre, London, Ontario N6A 4L6, Canada

## Abstract

**Background:**

Tasks chosen to evaluate motor performance should reflect the movement deficits characteristic of the target population and present an appropriate challenge for the patients who would be evaluated. A reaching task that evaluates impairment characteristics of people with shoulder impingement syndrome (SIS) was developed to evaluate the motor performance of this population. The objectives of this study were to characterize the reproducibility of this reaching task in people with and without SIS and to evaluate the impact of the number of trials on reproducibility.

**Methods:**

Thirty subjects with SIS and twenty healthy subjects participated in the first measurement session to evaluate intrasession reliability. Ten healthy subjects were retested within 2 to 7 days to assess intersession reliability. At each measurement session, upper extremity kinematic patterns were evaluated during a reaching task. Ten trials were recorded. Thereafter, the upper extremity position at the end of reaching and total joint excursion that occurred during reaching were calculated. Intraclass correlation coefficient (ICC) and minimal detectable change (MDC) were used to estimate intra and intersession reliability.

**Results:**

Intrasession reliability for total joint excursion was good to very good when based on the first two trials (0.77<ICC<0.99), and very good when based on either the first or last five trials (ICC>0.92). As for end-reach position, intrasession reliability was very good when using either the first two, first five or last five trials (ICC>0.82). Globally, MDC were smaller for the last five trials. Intersession reliability of total joint excursion and position at the end of reaching was good to very good when using the mean of the first two or five trials (0.69<ICC<0.95), and very good when using the mean of the ten trials (ICC>0.82). For most joints, MDC were smaller when using all ten trials.

**Conclusions:**

The reaching task proposed to evaluate the upper limb motor performance was found reliable in people with and without SIS. Furthermore, the minimal difference necessary to infer a meaningful change in motor performance was determined, indicating that relatively small changes in task performance can be interpreted as a change in motor performance.

## Introduction

Physical impairments to the upper extremity can significantly affect the ability to perform daily life activities [1]. The evaluation of the motor performance using kinematic data is often performed in order to establish the impact of the physical impairments on function [2,3]. The tasks chosen to evaluate motor performance should reflect the movement deficits characteristic of the target disorder and present an appropriate challenge for the spectrum of patients who would be evaluated. For example, people with shoulder impingement syndrome (SIS) present alterations in the movement of the scapula (increased or decreased scapular posterior tilting and lateral rotation) [4-6], humeral head (superior displacement with respect of the glenoid) [7] and clavicle (increased elevation and retraction) [6,8] during arm elevation. These movement deficits are most likely associated with a reduction of the subacromial space [9,10] leading to impingement of the subacromial structures [3]. The subacromial space reduction is more pronounced while performing arm elevation in the frontal plane as compared to the sagittal plane, thus leading to higher demands for shoulder control [11]. Therefore, the frontal plane is an optimal test position to appropriately challenge this target population [8].

Ideally, tasks used to evaluate motor performance should reflect activities of daily living. Most daily life activities require coordinated multi-joint movement of the upper extremity that balances stability and mobility while optimizing a goal oriented movement pattern. Unfortunately, the evaluation of the motor performance in people with SIS has been confined to simple arm elevation movements. Therefore, the upper extremity of people with SIS needs to be evaluated during multiarticular functional tasks in order to better represent the motor performance required during daily life activities [8].

Functional tasks, such as reaching out/pointing to targets and reaching for objects, have been used to evaluate the motor performance of people with physical impairments. For example, kinematic analysis of reaching has been used as an evaluative measure of upper extremity motor performance in people with shoulder dysfunctions [12] and in people after a stroke [13,14]. Two groups have evaluated the reliability of such reaching tasks and have reported favourable results [12,14]. Lin et al. [12] evaluated the within session reliability of shoulder girdle kinematics during functional tasks, including reaching tasks. They found intraclass correlation coefficients (ICC) values ranging from 0.73 to 0.99 and standard error of measurement (SEM) equal to or less than 2.5° for subjects with and without shoulder disorders. Wagner et al. [14] evaluated the within session reliability of forward reaching tasks in subjects with hemiparesis after stroke. Depending on the kinematic variable and the demands of the motor task, they observed ICC ranging from 0.04 to 0.99, and minimal detectable change ranging from 7.4% to 98,9%.

A reaching task that evaluates impairment characteristics of people with SIS was developed to evaluate the motor performance of this population [8]. However, the psychometric properties for evaluating longitudinal change in motor performance have not been established for this reaching task. The objective of this study was to characterize the reproducibility (ICC and SEM) and minimal detectable change (MDC) for reaching kinematics in people with and without SIS. A second objective was to evaluate the impact of the number of trials on reproducibility.

## Methods

### Subjects selection

Thirty subjects with primary subacromial SIS (mean age 47.9 years; 20 women, 10 men) and twenty healthy subjects (mean age 46.6 years; 13 women, 7 men) voluntarily participated in the study. All subjects with SIS were screened by an orthopaedic surgeon to rule out calcifications, shoulder instability and rotator cuff tear. They were considered eligible if there was at least one positive finding in each of these categories [15]: 1) painful arc of movement during flexion or abduction; 2) positive Neer or Kennedy-Hawkins impingement signs; 3) pain on resisted lateral rotation, abduction or Jobe test. Other exclusion criteria were previous shoulder surgery, shoulder pain during neck movement and shoulder capsulitis. The healthy subjects had no history of pain, movement limitation or previous surgery to the shoulder and neck. All the participants read and signed an informed consent form. This study was approved by the Ethics Committee of the Quebec Rehabilitation Institute.

### Study design and experimental procedures

All 30 subjects with SIS and 20 healthy subjects participated in the first measurement session and contributed to the evaluation of intrasession reliability. Ten healthy subjects were retested within 2 to 7 days to assess intersession reliability (mean 3.5 days). The subjects with SIS were participating in an intervention study following the first measurement session; therefore it was impossible to retest them without any intervention between measurement sessions. Subjects have to remain stable in time in order to evaluate reliability. Evaluators were blinded to the data from the first session when retesting.

**Figure 1:**
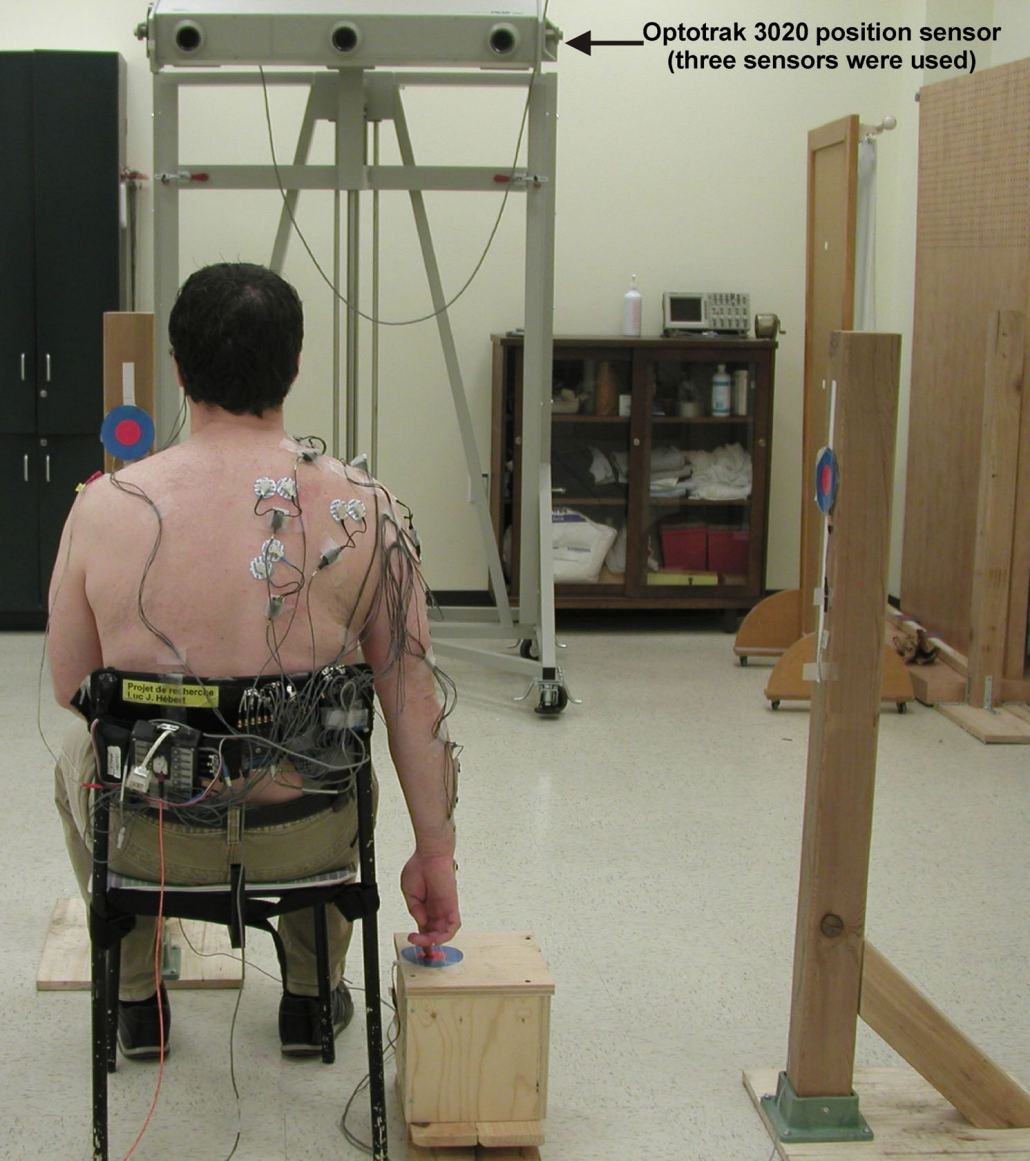
**Starting position for the reaching movement**. The reaching movements started with the upper extremity in a neutral position at the side of the body and the tip of the second finger in contact with a pressure switch. The kinematic was characterized using an optoelectric system and infrared light-emitting diodes positioned on five upper limb landmarks. As seen on the Figure, electromyography activity was also recorded, but the data were not analyzed in this study.

**Figure 2:**
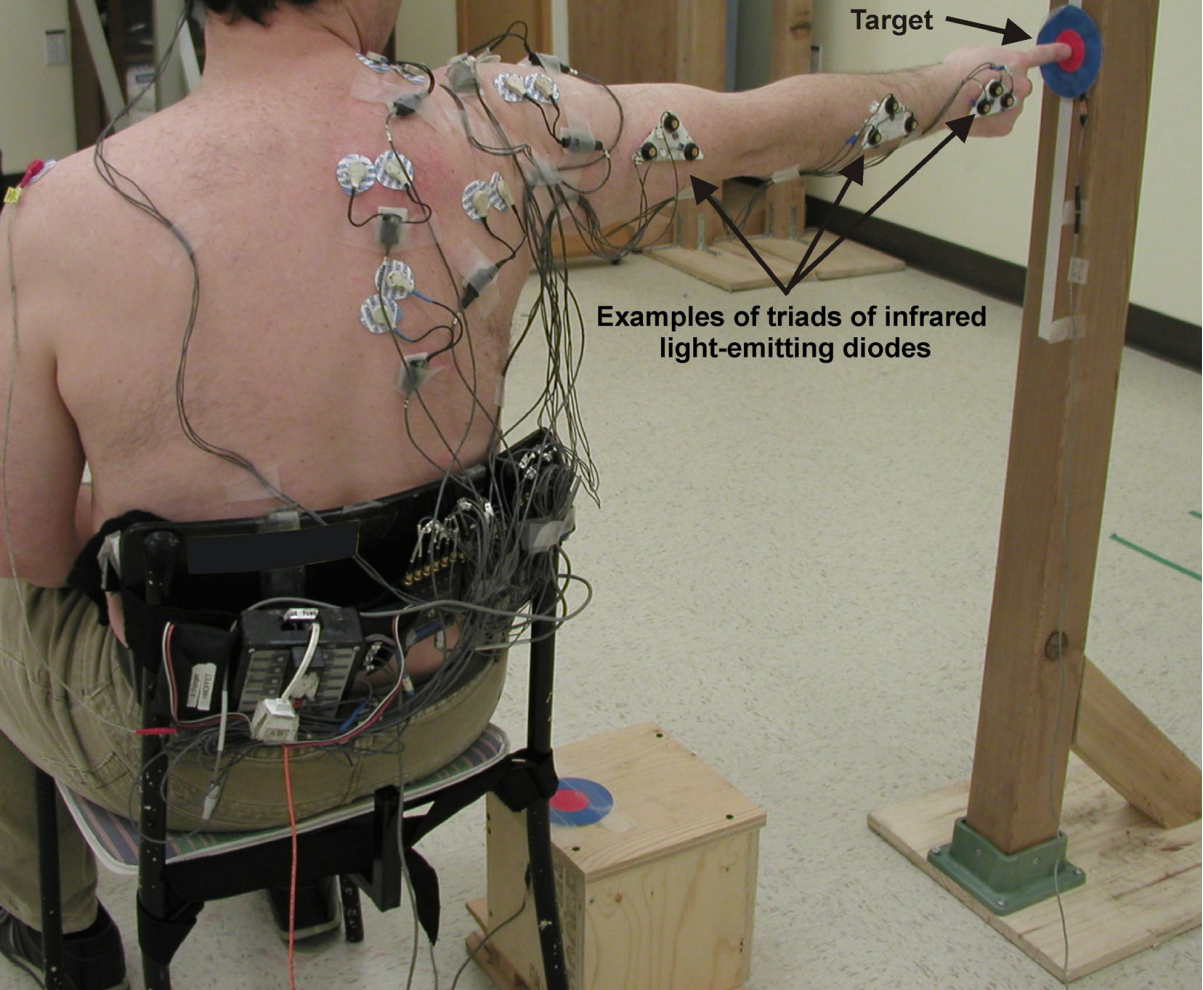
**Position at the end of reaching in the frontal plane**. The target was located in the frontal plane and positioned at a distance equivalent to the subject's arm length and at a height equivalent to the position of the second finger when the shoulder is at 90° of abduction. The kinematic was characterized using an optoelectric system and infrared light-emitting diodes positioned on five upper limb landmarks. As seen on the Figure, electromyography activity was also recorded, but the data were not analyzed in this study.

At each measurement session, the kinematic patterns of the upper extremity was evaluated during a tasks that consisted of reaching out and pointing (with contact) to a target. The subjects were seated with their knees and hips at 90°, their feet flat on the floor and their lumbar spine supported. The reaching movements started with the upper extremity in a neutral position at the side of the body and the tip of the second finger in contact with a pressure switch (Figure 1). An auditory cue signalled the beginning of the movement. The target was located in the frontal plane and positioned at a distance equivalent to the subject's arm length and at a height equivalent to the position of the second finger when the shoulder was at 90° of abduction (Figure 2). It was a round target and the diameter of its center (bull's-eye) was 4.5 cm (Figures 1 and 2). With their second finger, the subjects had to point the center of the target. A pressure switch was also placed under the center of the target to signal the end of reaching. Reaching was performed at a natural speed, as if the subjects were performing daily life activities. Since it was necessary to have an end signal to analyse the data, if subjects overshot or did not touch the center of the target, the trial was cancelled and repeated afterward. The movement was practiced three times before data acquisition. Ten trials were recorded. A rest period of 30 seconds was given after each trial. The symptomatic arm was evaluated for the subjects with SIS. For the healthy subjects, the side was chosen to have the same proportion of dominant/non-dominant sides as evaluated in the SIS group.

### Measurements

The upper extremity kinematics were characterized by the patterns of relative joint angles for the hand, elbow, shoulder, clavicle, and trunk. The Optotrak System (Northern Digital inc., Waterloo, Ontario, Canada) is an optoelectric system that was used to collect the 3-dimensional kinematic data of the upper extremity and the trunk (Figure 1). Three Optotrak 3020 position sensors were used. Triads of infrared light-emitting diodes were positioned on the hand (dorsal face), forearm (proximal to the styloid process of the radius), upper-arm (near the insertion of the deltoid), clavicle (lateral part of the clavicle) and trunk (top of the sternum) (Figure 2). A sampling rate of 100 Hz was used and data were digitally low-pass filtered at 8 Hz. As seen on Figures 1 and 2, the electromyography (EMG) activity of seven shoulder muscles was also recorded during the reaching task. However, the data of the EMG activity were not analyzed for this study.

Fourteen bony landmarks were digitized before the acquisition of data to recreate the coordinate systems [16]: C7 and T8 spinous processes, suprasternal notch and xiphoid process for the trunk; most ventral point on the sternoclavicular joint and most dorsal point on the acromioclavicular joint for the clavicle; most caudal point on the lateral and medial epicondyles for the humerus; root of the spine, inferior angle, acromial angle and most ventral point of processus coracoideus for the scapula (in order to estimate the glenohumeral rotation center by regression analysis [17]); and, most caudal-lateral and caudal-medial points on the radial and ulnar styloids for the forearm. Local coordinate systems and joint rotations were defined according to the Interntional Society of Biomechanics (ISB) recommendations [16]. The ISB recommends using the Grood and Suntay's convention to calculate joint movements of the upper extremity, except for the shoulder where a Cardanic *y-x-y *rotation matrix sequence was used [18].

In order to compare measurement, two periods (auditory cue to beginning of the movement and beginning to end of the movement) of 100 points were defined for the kinematics, with each point representing one percent of the period. Movement amplitudes were plotted for two degrees of freedom (DF) of the wrist (hand relative to the forearm: flexion/extension; deviation), one DF of the elbow (forearm relative to the arm: flexion/extension), three DF of the shoulder (humerus relative to the trunk: plane of elevation; elevation; rotation), two DF of the sternoclavicular joint (clavicle relative to the trunk: retraction/protraction; elevation/depression) and three DF of the trunk (trunk relative to the global system: flexion/extension; rotation; lateral flexion) [16]. Thereafter, the upper extremity angular position at the end of reaching and total joint excursion that occurred during reaching (difference between minimum and maximum absolute angles) were calculated in degrees.

### Data analysis

The reliability of the joint position at the end of reaching and of the total excursion was calculated for each joint evaluated. For the healthy and SIS subjects, the level of intrasession reliability was analyzed by comparing the first two trials, the first five trials and then, the last five trials. For the healthy subjects, the level of intersession reliability was analyzed by comparing the mean of the first two trials of the first session to the mean of first two trials of the second session. The same intersession comparison was done for the means of the respective first five trials and ten trials. The relative reliability was estimated by calculating the intraclass correlation coefficients (ICCs) and its 95% confidence interval (95% CI) [19,20]. ICCs values were considered to reflect: a poor reliability when below 0.20; a fair reliability from 0.21 to 0.40; a moderate reliability from 0.41 to 0.60; a good reliability from 0.61 to 0.80 and, a very good reliability from 0.81 to 1.00 [21]. The absolute reliability was calculated with standard errors of measurement (SEM) and its 95%CI, and minimal detectable change (MDC) [22]. The MDC was calculated by multiplying the *z*-score corresponding to the level of significance, the square root of 2, and the SEM [23]. A *z*-score of 1.65 was chosen to reflect an acceptable 90% confidence level for clinical application to individual patients [23]. Significant differences in reliability between groups and between numbers of trials were determined when the 95% CI of the ICC or the SEM were not overlapping. All analyses were conducted with the SPSS software (Version 12; SPSS Inc, 233 S Wacker Dr, 11th Fl, Chicago, IL 60606). The alpha level was set at 0.05.

## Results

**Figure 3:**
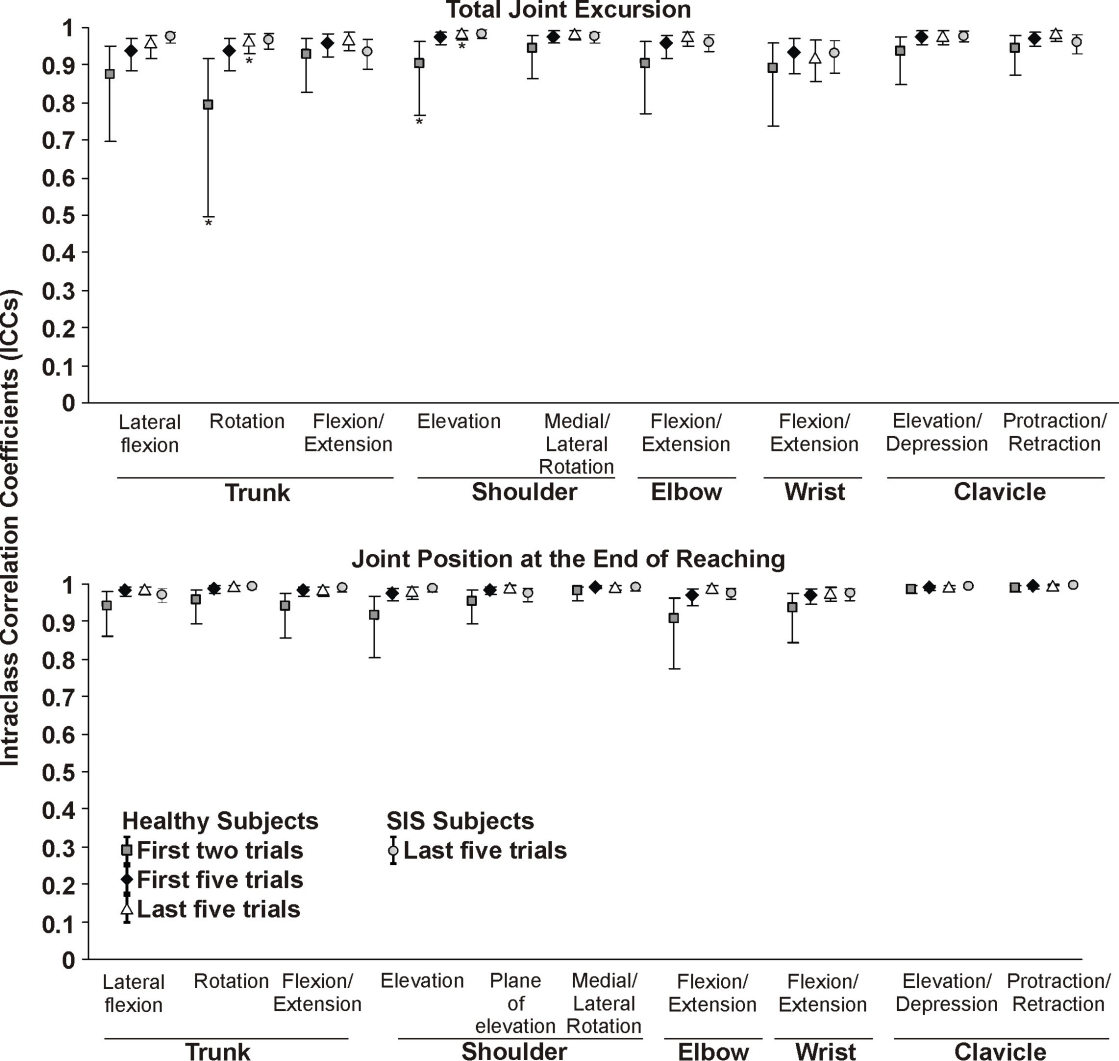
**Intrasession intraclass correlation coefficients (ICC)**. The ICCs and its 95% confidence interval are presented for the first two trials, the first five trials and the last five trials out of ten in healthy subjects (n = 20) and for the last five trials in subjects with SIS (n = 30). The ICC are only presented for the last five trials in the SIS groups since the ICC were similar for the subjects with and without SIS. * Significant differences (*P *< 0.05) between the first two trials and the last five trials for healthy subjects.

**Figure 4:**
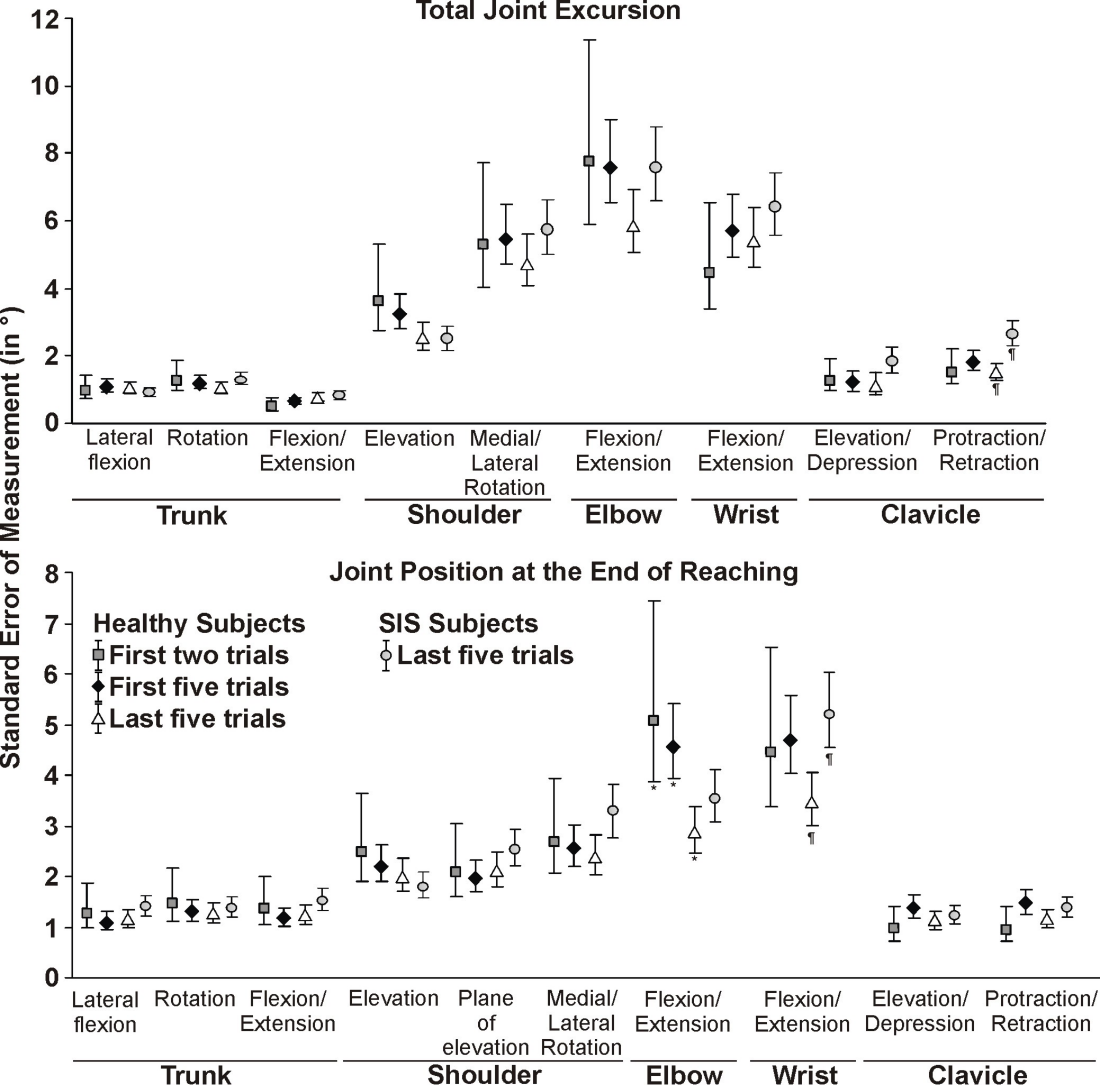
**Intrasession standardized error of measurement (SEM)**. The SEM and its 95% confidence interval are presented for the first two trials, the first five trials and the last five trials out of ten in healthy subjects (n = 20) and for the last five trials in subjects with SIS (n = 30). The SEM are only presented for the last five trials in the SIS groups since the SEM were similar for most joints in subjects with and without SIS. * Significant differences (*P *< 0.05) for the first two trials and the first five trials compared to the last five trials for healthy subjects. ^¶^Significant differences (*P *< 0.05) between healthy subjects and the subjects with SIS.

Within the same session, the reliability of scores based on the first two trials of upper extremity total joint excursion was good to very good for both patients and asymptomatic subjects and very good when using either the first five or last five trials (Figures 3 and 4; Table 1). There was a trend towards higher reliability coefficients when within session reliability was based on five trials. However, the ICC was only significantly higher when using the last five trials compared to the first two trials for trunk rotation and shoulder elevation for the healthy group (Figure 3). There were no significant differences in the level of reliability between the subjects with and without SIS, except for clavicular protraction/retraction for which the SEM were smaller in the healthy group (Figure 4).

**Table 1:** Intrasession reliability of upper extremity reaching movement: last five trials out of ten.

Joint	Movement		Control Group (n = 20)				SIS Group (n = 30)			
				
			ROM	ICC	SEM	MDC 90%	ROM	ICC	SEM	MDC90%
Trunk	Lateral flex	Excursion	4.9°	0.96	1.1°	2.4°	5.2°	0.98	0.9°	2.0°
		Final position	2.0°	0.99	1.1°	2.7°	1.7°	0.98	1.1°	2.7°
	Rotation	Excursion	5.2°	0.96	1.0°	2.4°	8.0°	0.96	1.5°	3.4°
		Final position	10.3°	0.99	1.2°	2.9°	15.4°	0.99	1.3°	3.0°
	Flex/Ext	Excursion	2.4°	0.97	0.8°	1.8°	2.9°	0.93	0.8°	2.0°
		Final position	0.2°	0.98	1.2°	2.8°	0.5°	0.99	1.2°	2.8°
S/C	Ele/Dep	Excursion	8.3°	0.98	1.1°	2.6°	10.2°	0.95	2.9°	6.9°
		Final position	18.0°	0.99	1.1°	2.6°	20.6°	0.99	0.9°	2.2°
	Pro/Ret	Excursion	14.9°	0.98	1.5°	3.4°	15.4°	0.92	4.5°	10.4°
		Final position	34.0°	0.99	1.1°	2.7°	35.4°	0.99	1.2°	2.8°
Shoulder	Elevation	Excursion	77.8°	0.98	2.5°	5.9°	78.1	0.99	2.1°	4.9°
		Final position	77.5°	0.98	2.0°	4.6°	78.4°	0.99	1.9°	4.3°
	Plane*	Excursion	N/A	N/A	N/A	N/A	N/A	N/A	N/A	N/A
		Final position	4.9°	0.99	2.1°	4.9°	9.2°	0.99	1.7°	4.0°
	Rotation	Excursion	65.2°	0.98	4.7°	11.0°	76.6	0.97	5.2°	12.1°
		Final position	51.7°	0.99	2.4°	5.5°	50.3°	0.99	2.7°	6.2°
Elbow	Flex/Ext	Excursion	31.4°	0.97	5.9°	13.6°	25.7°	0.98	5.9°	13.7°
		Final position	30.6°	0.99	2.8°	6.6°	30.5°	0.97	3.9°	8.8°
Wrist	Flex/Ext	Excursion	41.1°	0.92	5.4°	12.5°	41.8°	0.94	5.9°	13.7°
		Final position	12.6°	0.98	3.3°	7.7°	12.4°	0.98	4.2°	9.9°

Abbreviations: S/C, sternoclavicular joint; Flex, Flexion; Ext, Extension; Plane, Plane of elevation; Ele, Elevation; Dep, Depression; Pro, Protraction; Ret, Retraction.

* Shoulder plane of elevation was only calculated for the total joint excursion

The intrasession reliability was very good for end-reach position when using either the first two trials, first five trials or last five trials. However, for some joints, the 95%CI was larger when using the first two trials. Significant differences were only observed for elbow flexion/extension in the healthy group, where the SEM were lower when using the last five trials compared to the first two or five trials (Figure 4). Globally, the SEM and MDC were smaller for the last five trials compared to the first two or five trials. There were no significant differences between the subjects with and without SIS, except for wrist flexion/extension for which the SEM were smaller in the healthy group (Figure 4).

**Figure 5:**
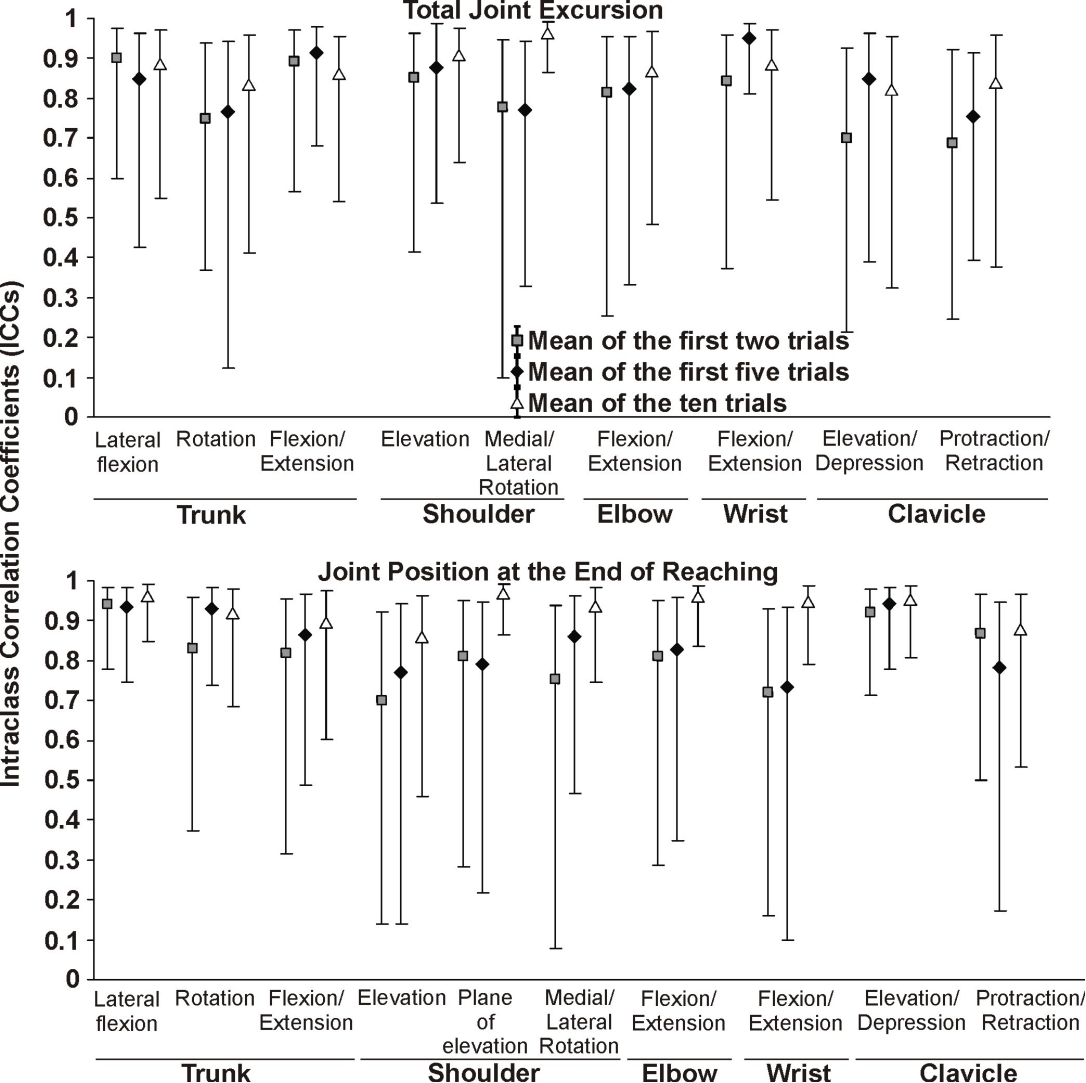
**Intersession intraclass correlation coefficients (ICC)**. The ICCs and its 95% confidence interval are presented for the mean of the first two trials, the first five trials and all ten trials in healthy subjects (n = 10).

**Figure 6:**
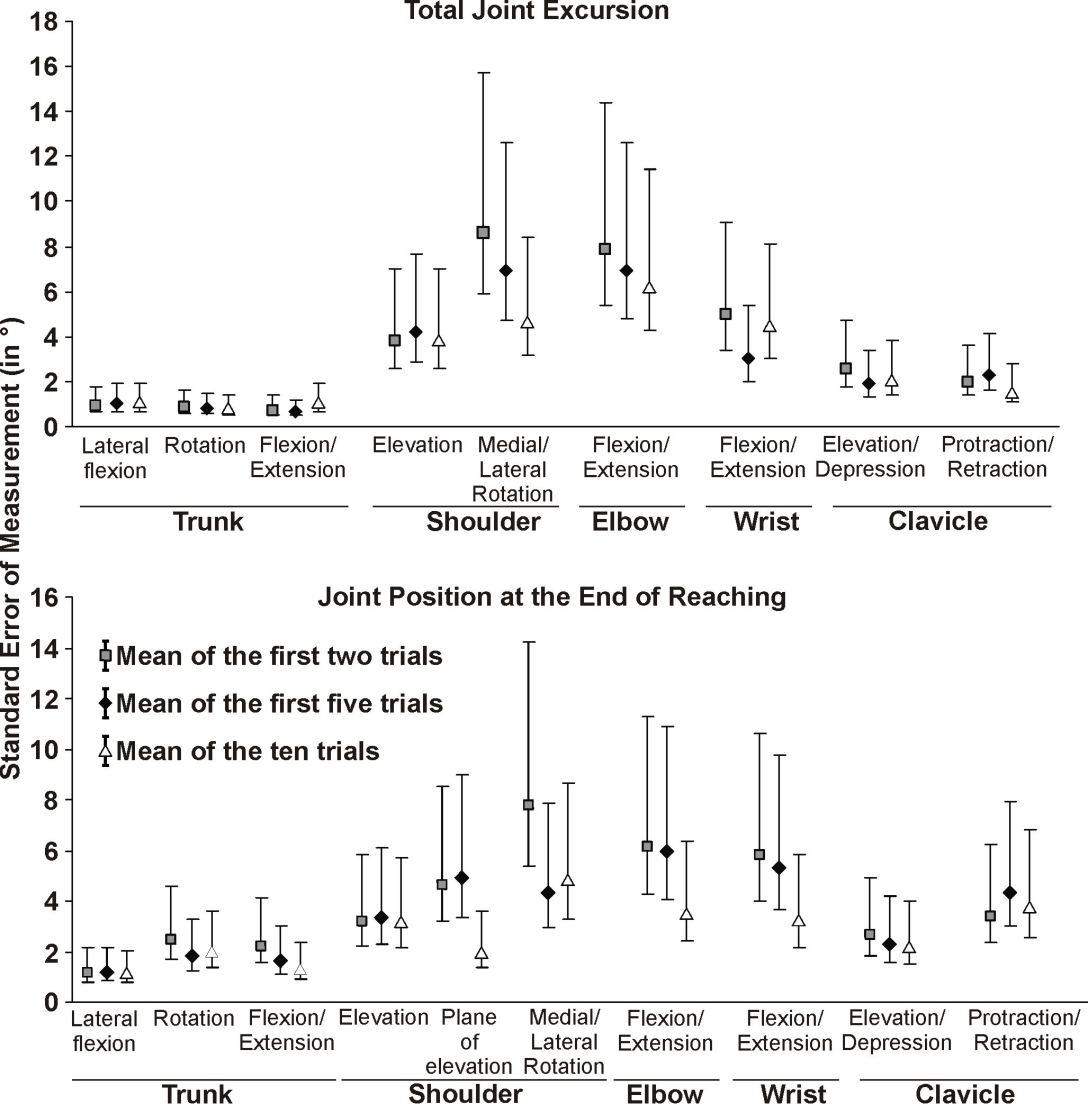
**Intersession standardized error of measurement (SEM)**. The SEM and its 95% confidence interval are presented for the mean of the first two trials, the first five trials and all ten trials in healthy subjects (n = 10).

Between session (test-retest), reliability of upper extremity total joint excursion was good to very good in healthy subjects when using the mean of the first two or five trials, and very good when using the mean of the ten trials (Figure 5 and 6; Table 2). As for the intersession reliability for the upper extremity position at the end of reaching, the intersession reliability was again good to very good when using the mean of the first two or five trials, and very good when using the mean of the ten trials (Figure 5 and 6; Table 2). For most of the joints, the range of the 95%CI and the MDC were smaller when using all ten trials, especially for the position at the end of reaching. However, it did not result in significant differences in reliability for both the total joint excursion and the joint position at the end of reaching.

**Table 2:** Intersession reliability of upper extremity reaching movement: mean of ten trials.

			Control Group (n = 10)			
			
Joint	Movement		ROM^¶^	ICC	SEM	MDC90%
Trunk	Lateral flex	Excursion	6.1°	0.88	1.0°	2.4°
		Final position	3.5°	0.96	1.1°	2.6°
	Rotation	Excursion	5.1°	0.83	0.8°	1.8°
		Final position	12.2°	0.92	2.0°	4.6°
	Flex/Ext	Excursion	2.4°	0.82	1.0°	2.4°
		Final position	1.6°	0.90	1.3°	3.0°
S/C	Ele/Dep	Excursion	8.5°	0.82	2.1°	4.9°
		Final position	16.9°	0.95	2.2°	5.1°
	Pro/Ret	Excursion	13.6°	0.84	1.5°	3.6°
		Final position	31.9°	0.88	2.1°	4.9°
Shoulder	Elevation	Excursion	77.9°	0.90	3.8°	8.9°
		Final position	77.7°	0.86	3.1°	7.3°
	Plane*	Excursion	N/A	N/A	N/A	N/A
		Final position	4.7°	0.97	2.0°	4.6°
	Rotation	Excursion	67.1°	0.96	4.6°	10.7°
		Final position	47.4°	0.93	4.8°	11.1°
Elbow	Flex/Ext	Excursion	25.7°	0.86	6.2°	14.5°
		Final position	30.7°	0.96	3.5°	8.1°
Wrist	Flex/Ext	Excursion	40.1°	0.88	4.4°	10.3°
		Final position	18.0°	0.95	3.2°	7.4°

Abbreviations: S/C, sternoclavicular joint; Flex, Flexion; Ext, Extension; Plane, Plane of elevation; Ele, Elevation; Dep, Depression; Pro, Protraction; Ret, Retraction.

* Shoulder plane of elevation was only calculated for the finishing joint position.

^¶ ^From the second measurement session.

## Discussion

This study established that kinematic parameters of a functional task can be consistently performed by patients with SIS or asymptomatic subjects within a single session and by asymptomatic subjects across a test-retest period. It was also established that MDC could be used as a benchmark for clinically relevant differences when evaluating a patient following an intervention.

The intra- and intersession reliability statistics indicate that ideally, five to ten trials should be performed depending if intra- or intersession evaluations are performed. If only intrasession evaluation is needed, either the first or last five trials should be used to establish stable results. As for intersession reliability, using the mean of 10 trials did not result in better reliability coefficients but did define smaller absolute error estimates so that smaller minimal detectable change could be identified. The majority of time required for this testing is for the set up procedures (positioning of the markers, probing bony landmarks, calibration). The actual reaching task is fairly quick and easy for most participants. Therefore, an additional five repetitions to reduce test-retest measurement error is worthwhile. However, if patients experience high level of pain during the reaching task, then fewer repetitions should be performed. If fewer repetitions are performed, then changes in time have to be analyzed in light of higher absolute error estimates. Lin et al. [12] also found similar findings for reaching tasks in that the measurement error decreases when the number of trials increased.

The coordination of the multiple degrees of freedom available during the reaching tasks could explain the need for multiple trials in order to have good repeatability with acceptable confident intervals. Reaching implies the coordination of numerous upper extremity joints. Coordination of movement is the process of mastering the redundant degrees of freedom in order to have a controllable system [1]. As a result, a given movement can be performed using different kinematic and kinetic patterns [24]. By providing a standardized starting position and a target for the final position, a standardized task goal was created. Although providing a target for the final position may seem structured, many functional movements like taking objects off of shelves or pressing an elevator button require reaching with a specific end target position. Furthermore, the subjects retained a large amount of control and potential variability in how that goal is executed. Since this specific targeted task is new to the subject, several trials may need to be executed before the subject develops a consistent strategy and becomes comfortable with the execution of the task. In the present study only three practice trials were performed before the recording. The data suggest that additional practice trials might improve repeatability. The fact that a target was provided may explain why end-reach position was more consistent than total joint excursion. Different combinations of kinematic and kinetic patterns can be used to get to the target. However, these patterns result in a final joint position that is more constant across trials.

Reliability within sessions was not statistically different between patients and healthy subjects, although there was a trend for the healthy subjects to be more consistent. Pain may have interfered to some extent with the consistency of the performance of patients with SIS. The plane chosen to execute the task and the position of the target were selected to challenge patients with SIS, so this effect was expected. The pain level was evaluated after each trial for the subjects with SIS using the Present Pain Index [25]. The mean pain level was low at 0.9 out of 5 (Standard deviation = 0.8), but all the subjects experienced pain during the evaluation session. Patients experiencing pain can be expected to try different movement strategies in order to reduce the pain arising from impingement of the subacromial structures under the coracoacromial arch.

To determine whether change in the motor performance is meaningful, the MDC values can be used [26]. For example, if the same patient who had a total excursion in clavicular elevation of 14° on the initial evaluation has an end-reach position in clavicular elevation of 8.5° during reassessment 2 weeks later, the clinician will be able to state confidently that the patient has demonstrated statistically meaningful improvement because the change of 5.5° is greater than the MDC value (4.9°). In fact, in a previous study, a difference of 6.3° was observed for total excursion in clavicular elevation between a subgroup of subjects with SIS and healthy subjects [8]. Following a single session of rehabilitation, a reduction of 2.8° in clavicular excursion was observed. The change was statistically significant, but still not meaningful. MDC can be used to set short to mid term measurable treatment goals.

A number of limitations should be considered when interpreting the results. First, the current proposed method cannot be generalized to reaching in other planes of movement since the reliability has only been tested for a target located in the frontal plane. Second, the intersession reliability was only evaluated in a subgroup of healthy subjects. This could explain the large variations in the 95%CI obtained for the between session measurements and the magnitude of the MDC for some variables (mostly the more distal joints-elbow and wrist). Finally, a skin-based method, which involves digitizing bony landmarks and infrared-emitting diodes, was used for measuring the upper-extremity kinematics. Therefore, skin motion artefacts may affect data accuracy[12]

## Conclusion

The reaching task proposed to evaluate the motor performance of people with SIS was found reliable in people with and without SIS. Furthermore, the minimal difference necessary to infer a meaningful change in motor performance was determined, indicating that relatively small changes in task performance can be interpreted as a change in motor performance. This reaching task may now be used to characterize changes in the motor performance of people with SIS. In future studies, it will be important to analyze the effects of medical/rehabilitation interventions on the motor performance in light of the minimal detectable change.

## List of abbreviations

ANOVA: Analysis of variance; CI: Confidence interval; DF: Degrees of freedom; ICC: Intraclass correlation coefficient; ISB: International Society of Biomechanics; MDC: Minimal detectable change; SEM: Standard error of measurement; SIS: Shoulder impingement syndrome; EMG: Electromyographic.

## Competing interests

The authors declare that they have no competing interests.

## Authors' contributions

JSR: participated in the design of the study, carried out the acquisition, the analysis and the interpretation of data and drafted the manuscript. HM: participated in the design of the study, the analysis and the interpretation of data and drafted the manuscript. BJM: participated in the design of the study, carried out the acquisition, the interpretation of data and drafted the manuscript. JCM: participated in the development of the study question, the analysis and the interpretation of data and drafted the manuscript. All authors read and approved the final manuscript.
